# « A reason why » : Suicide attempt after Coronavirus infection

**DOI:** 10.1192/j.eurpsy.2022.1320

**Published:** 2022-09-01

**Authors:** R. Jomli, H. Jemli, H. Ghabi, A. Aissa, U. Ouali

**Affiliations:** 1 Razi Hospital, Psychiatry A, manouba, Tunisia; 2 university of tunis elmanar, Faculty Of Medicine Of Tunis, manouba, Tunisia

**Keywords:** Anxiety, COVID19, Distress, Suicide

## Abstract

**Introduction:**

COVID19 pandemic had an important emotional and psychological impact due to the higher rates of boredom, fear, stress, anxiety, depression, etc. (Brooks et al., 2020). Cases of suicide during the COVID-19 pandemic are increasingly reported. According to an Indian study, the main cause of suicide was fear or anticipation of COVID-19 infection.

**Objectives:**

To illustrate a case of suicide attempt after COVID-19 infection.

**Methods:**

We report the case of a Tunisian man who did a suicide attempt after his infection with the coronavirus.

**Results:**

A 35-year-old Tunisian man, married, an official, with no medical or psychiatric history who was admitted in the Oto-rhino-laryngology department, after a suicide attempt by strangulation, five days after the diagnosis of COVID-19 infection. In fact, the patient was very stressed about his infection and feared transmitting the virus to his family. During his quarantine, he became anxious, had insomnia and suicidal thoughts. He was isolated in his room, and prohibit his family to approach his room. He told us that he could not support this anxiety and he decided to suicide to end this situation and save his family.

**Conclusions:**

The COVID-19 pandemic may increase suicide rates. Psychological consequences of this pandemic including suicide may continue to incur later than this actual worldwide crisis. Mental health promotion is the key to prevent and mitigate such mental health consequences.

**Disclosure:**

No significant relationships.

